# Hyperglycemia Determines Increased Specific MicroRNAs Levels in Sera and HDL of Acute Coronary Syndrome Patients and Stimulates MicroRNAs Production in Human Macrophages

**DOI:** 10.1371/journal.pone.0161201

**Published:** 2016-08-12

**Authors:** Natalia Simionescu, Loredan S. Niculescu, Mihaela G. Carnuta, Gabriela M. Sanda, Camelia S. Stancu, Andreea C. Popescu, Mihaela R. Popescu, Adelina Vlad, Doina R. Dimulescu, Maya Simionescu, Anca V. Sima

**Affiliations:** 1 Lipidomics Department, Institute of Cellular Biology and Pathology “Nicolae Simionescu” of the Romanian Academy, Bucharest, Romania; 2 Centre of Advanced Research in Bionanoconjugates and Biopolymers, “Petru Poni” Institute of Macromolecular Chemistry, Iasi, Romania; 3 Cardiology Clinic, Elias University Emergency Hospital, Bucharest, Romania; 4 Physiology Department, “Carol Davila” University of Medicine and Pharmacy, Bucharest, Romania; National Institutes of Health, UNITED STATES

## Abstract

We aimed to determine the levels of microRNAs (miRNAs) in sera and HDL of acute coronary syndrome (ACS) compared to stable angina (SA) patients with/without hyperglycemia, and evaluate comparatively the functional effect of these sera on the processing machinery proteins (Drosha, DGCR8, Dicer) and miRNAs production in human macrophages. MiRNAs levels in sera and HDL from 35 SA and 72 ACS patients and 30 healthy subjects were measured by using microRNA TaqMan assays. MiR-223, miR-92a, miR-486, miR-122, miR-125a and miR-146a levels were higher in the hyperglycemic ACS compared to normoglycemic sera. MiR-223 and miR-486 prevailed in HDL_2_, while miR-92a predominated in HDL_3_, all three miRNAs discriminating between ACS and SA patients; their levels were increased in HDL from hyperglycemic ACS patients versus normoglycemic ones. The incubation of human macrophages with sera from ACS and SA patients showed that all patients’ sera induced an increase of Drosha, DGCR8 and Dicer expressions and of selected miRNAs levels compared to control sera, the effect being higher in the case of hyperglycemic versus normoglycemic ACS sera. The addition of glucose to SA and ACS sera increased Drosha, DGCR8 and Dicer expression and miRNAs levels in the exposed macrophages. In conclusion, hyperglycemia is associated with increased miR-223, miR-92a, miR-486 levels in HDL, which discriminate between ACS and SA patients. Exposure of human macrophages to ACS compared to SA sera determines the upregulation of Drosha, DGCR8 and Dicer expression and the increase of selected miRNAs production, the effect being augmented by an increased glucose concentration.

## Introduction

MicroRNAs (miRNAs) are small non-coding RNAs that act as gene regulators by inhibiting translation [1, 2]. MiRNAs are transcribed by RNA polymerase II as pri-miRNAs [3] and are further processed to pre-miRNAs by the microprocessor complex comprised of the RNase III enzyme Drosha bound by its regulatory subunit DGCR8 [4, 5]. The pre-miRNAs are then transported to the cytoplasm by Exportin-5 [6], where they are cleaved to miRNA duplex intermediates by the RNase III enzyme, Dicer [7]. Then, the leading miRNA strand is selected and loaded into Argonaute proteins and they regulate together the expression of target genes downstream [7]. MiRNAs can be exported outside the cells, circulate in the blood associated with microparticles, exosomes, lipoproteins (Lp) or protein complexes and act as long-distance extracellular messengers [8–11]. Modified cellular expression of miRNAs or altered circulating miRNAs profiles have been associated with several diseases, including atherosclerosis, obesity, diabetes and coronary artery disease [12–16].

Atherosclerosis is the major cause of cardiovascular diseases (CVD) [17] and of morbidity and mortality worldwide. Atherosclerotic plaques development in the wall of coronary arteries results in coronary artery disease (CAD). In the first stages of plaque formation, endothelial cells become activated and turn toward a secretory phenotype, leading to the development of a hyperplasic basal lamina and recruitment of inflammatory cells [18]. Circulating monocytes migrate into the subendothelium and differentiate into macrophages, becoming the hallmark of the atherosclerotic plaque [17]. Serum proteins, atherogenic Lp, such as low density Lp (LDL), and anti-atherogenic Lp, such as high density Lp (HDL), reach the subendothelium by transcytosis through the endothelial cells [19]. In the hyperplasic basal lamina and extracellular matrix, they accumulate, suffer modifications and interact with the macrophages, leading to lipid-loading and foam cell-formation [18]. The progression and gravity of the atherosclerotic plaque is difficult to evaluate and therefore it is important to elaborate non-invasive methods to assess the evolution of acute coronary syndromes (ACS).

In this study, we evaluated the levels of a panel of six miRNAs (miR-223, miR-92a, miR-486, miR-122, miR-125a and miR-146a) in sera and HDL from stable angina (SA) and ACS patients, and the functional effects of ACS and SA patients’ sera, with or without hyperglycemia, on cultured human macrophages, namely on the gene expression of the processing machinery proteins (Dicer, Drosha, DGCR8) and analyzed miRNAs production. It is generally accepted that hyperglycemia is an accelerating factor for the evolution of CAD [20], so we aimed to estimate the effect of increased glucose on the selected miRNAs production in macrophages.

## Material and Methods

### Study design and subjects

The investigation included 137 subjects (59 women and 78 men, aged 24–79 years): 107 patients (34 women and 73 men, aged 35–79 years) with CAD (35 SA and 72 ACS), with or without hyperglycemia and 30 healthy control subjects (25 women and 5 men, aged 24–62 years). All CAD patients were recruited from the Cardiology Clinic, Elias Emergency University Hospital, Bucharest, between November 2012 and December 2015. Control subjects were healthy donors recruited from the staff of the Institute of Cellular Biology and Pathology “Nicolae Simionescu” and of the Elias Hospital, without CVD risk factors or other documented disorders. Patients with autoimmune or malignant diseases, acute infections and severe hepatic or renal diseases were excluded from the study.

Inclusion in SA and ACS groups was done according to the guidelines of the European Society of Cardiology, by clinical assessment, cardiac biomarkers levels, electrocardiography and echocardiography. SA patients presented typical angina, history of CAD and/or positive electrocardiography stress test. ACS patients had unstable angina, with or without a history of myocardial infarction, as defined by the guidelines of the European Society of Cardiology and treated in the Cardiology Clinic of Elias Hospital. This study was performed respecting the principles in the Declaration of Helsinki (The Code of Ethics of the World Medical Association, updated at the 64th WMA General Assembly, Fortaleza, Brazil, October 2013) for experiments involving humans. All participants gave their written informed consent. The Ethics Committees of the Institute of Cellular Biology and Pathology “Nicolae Simionescu” and of the Elias University Emergency Hospital have approved the study.

Serum was isolated from fasting blood samples of each patient and biochemical and miRNAs analysis were performed. None of the patients received heparin or fractionated heparin at the time of sampling. For serum Lp isolation, equal amounts of sera from patients of each group were pooled, with respect to the presence or absence of hyperglycemia; the procedure was performed on three independent pools of sera from each group.

### Determination of serum parameters

Serum total cholesterol (TC), triglycerides (TG) and fasting glucose were measured in all subjects and patients using automated biochemical analyzers. Non-esterified fatty acids (NEFA) were determined by end-point colorimetric method (Wako Chemicals GmbH, Neuss, Germany), HDL cholesterol (HDL-C) and LDL cholesterol (LDL-C) by commercially available kits (Dialab Gmbh., Neudorf, Austria), apolipoproteins A-I (apoA-I), apoB-100 and apoE by commercial enzyme-linked immunosorbent assay (ELISA) kits (Mabtech AB, Nacka Strand, Sweden). The paraoxonase 1 (PON1) activity was determined using an adapted method described by Rozenberg et al. [21].

### Isolation of serum lipoproteins

We isolated high-density lipoproteins (HDL), with their subpopulations (HDL_2_ and HDL_3_) from the group-pooled sera by a previously reported method [22]. After that, 20 fractions (0.5 mL each) were collected from each tube and dialyzed against phosphate-buffered saline (PBS) pH 7.4, at 4°C in the dark. The protein in the collected fractions was assessed by a modified Lowry method [23]. Fractions in each group corresponding to HDL_2_ and HDL_3_ subpopulations were pooled and kept at -80°C until miRNAs analysis.

### Analysis of miRNAs in sera and lipoproteins

MiRNeasy Serum/Plasma kit (Qiagen, Dusseldorf, Germany) was used to isolate miRNAs from 200 μL sera and from 300 μL Lp fractions (120 μg protein), according to the manufacturer’s instructions. 25 fmol of synthetic *cel-miR-39* (Applied Biosystems, Thermo Fisher Scientific, Waltham, MA USA) was used as a spike-in to correct sample-to-sample variation, as previously described [22], RNA was eluted with 20 and 30 μL, respectively, of RNase-free water and then stored at -80°C.

The serum and Lp levels of *Homo sapiens* (hsa)-miR-223-3p (ID002295), hsa-miR-92a-3p (ID000431), hsa-miR-486-5p (ID001278), hsa-miR-122-5p (ID002245), hsa-miR-125a-5p (ID002198), hsa-miR-146a-5p (ID000468) and cel-miR-39-5p (ID000200) were determined by employing the TaqMan technology. Reverse-transcription was done with a pool of TaqMan miRNA-specific stem-loop primers on a Veriti PCR system (Applied Biosystems, Thermo Fisher Scientific). Real-time quantitative PCR was performed using the hydrolysis probes of miRNA TaqMan assays on a ViiA7 real-time PCR system (Applied Biosystems, Thermo Fisher Scientific) and for each sample, triplicate measurements were done on 384-well reaction plates. The data were analyzed using the ViiA7 Software v1.2 (Applied Biosystems, Thermo Fisher Scientific) with the automatic Cq setting. The level of each miRNA was determined relative to cel-miR-39 and calculated using the 2^-ΔCq^ method (ΔCq = Cq^hsa-miRNA^—Cq^cel-miR-39^) [24], multiplied by 10^6^ (for serum and Lp levels) and then log-transformed for the statistical analysis (for serum levels).

### Cell culture and experimental design

Human THP-1 monocytes (ATCC, Manassas, Virginia, USA) were grown in RPMI-1640 medium (Sigma-Aldrich, St. Louis, MO, USA) containing 10% heat-inactivated fetal calf serum (EuroClone, Siziano, Italy) and penicillin/streptomycin. For experiments, the monocytes were seeded at 10^6^ cells/ml density and differentiated into macrophages by exposure to 100 nM phorbol-12-myristate-13-acetate (Sigma-Aldrich, St. Louis, MO, USA) for 72 hours. The cells were then incubated for 24h with 10% human lipoprotein-deficient serum (LPDS) or with 10% serum from normoglycemic and hyperglycemic CAD patients. In some experiments, normoglycemic CAD sera were supplemented with 5.5 mM (100 mg/dl) glucose. After 24h incubation, the cells were rinsed once with PBS and total RNA extracted with TRIzol reagent (Ambion, Life Technologies, Thermo Fisher Scientific), according to manufacturer’s protocol. Macrophages were incubated with minimum 3 different sera from each group (CAD and control), the experiments being performed in triplicate.

### MiRNAs and gene expression analysis in THP-1 macrophages

Dual reverse-transcription of RNA was performed on a Veriti PCR system, using the High Capacity cDNA Reverse Transcription kit, random RT primers and a pool of TaqMan miRNA-specific stem-loop primers (all from Applied Biosystems, Thermo Fisher Scientific) for hsa-miR-223-3p (ID002295), hsa-miR-92a-3p (ID000431), hsa-miR-486-5p (ID001278), hsa-miR-122-5p (ID002245), hsa-miR-125a-5p (ID002198), hsa-miR-146a-5p (ID000468) and snRNU6 (ID001973).

Real-time quantitative PCR reactions were performed on a ViiA7 real-time PCR system (Applied Biosystems, Thermo Fisher Scientific) and for each sample triplicate measurements were done on 384-well reaction plates. The data were analyzed using the ViiA7 Software v1.2 (Applied Biosystems, Thermo Fisher Scientific) with the automatic Cq setting.

For miRNAs analysis, the real-time PCR was done using the hydrolysis probes of TaqMan miRNA assays and the Gene Expression Real-Time PCR Master Mix (both from Applied Biosystems, Thermo Fisher Scientific). The expression level of each miRNA was determined relative to snRNU6 and calculated using the 2^-ΔΔCq^ method [24].

For gene expression analysis, the real-time PCR was done using specific hydrolysis probes for Dicer, Drosha, DGCR8 and β-actin (Invitrogen, Thermo Fisher Scientific) and the SyBr Select Real-Time PCR Master Mix (Applied Biosystems, Thermo Fisher Scientific), according to the manufacturer’s instructions. The expression level of each gene of interest was determined relative to β-actin (as housekeeping gene) and calculated using the 2^-ΔΔCq^ method [24].

### Statistical analysis

The SPSS for Windows v21.0 (IBM SPSS, IBM Ireland, Dublin, Ireland) statistical software was used for statistical analysis. The continuous distributed quantitative variables (biochemical, mRNA and miRNA data) were expressed as means ± standard error of the mean (SEM) and analyzed by two-tailed Oneway ANOVA with *Least Significant Difference* (LSD) Post-hoc test (for the 3 groups of studied subjects, Control, SA and ACS) and Student T-test (for differences between normoglycemic and hyperglycemic subjects in each group). Crosstabs distribution with chi-squared (χ^2^) analysis was performed to evaluate the differences between binary data (gender, age distribution, presence of obesity, diabetes or hypertension, use of medication). The values obtained for circulating miRNAs levels in all patients’ sera were multiplied by 10^6^ and log-transformed. Parametric bivariate correlation analysis of log-transformed miRNAs levels with serum lipids and inflammation parameters were performed using the Pearson’s function and corresponding p-values. The threshold for statistical significance was set to 5% (p-values lower than 0.05).

## Results

### MiRNAs levels in CAD patients’ sera and HDL subfractions

The clinical data, serum lipids, apolipoproteins levels and paraoxonase 1 (PON1) activity of the hyperglycemic (HG) CAD patients, compared to the normoglycemic (NG) ones are presented in Tables 1 and 2. All CAD patients were under standard treatment with statins, aspirin, anti-platelets medication and ACE-inhibitors (Table 1). Consequently, they had significantly lower levels of TC, LDL-C, HDL-C, and PON1 activity, and higher TG and apoE levels, regardless of the presence of hyperglycemia (Table 2). Hyperglycemia was associated with significantly lower HDL-C levels and PON1 activity (p<0.05, Table 2) and significantly higher TG and NEFA levels in the respective CAD patients sera (p<0.01, Table 2).

**Table 1:** Clinical characteristics of the normoglycemic and hyperglycemic CAD patients and control subjects.

Parameter	Control (n = 30)		SA (n = 35)		ACS (n = 72)	
	NG (n = 27)	HG (n = 3)	NG (n = 27)	HG (n = 8)	NG (n = 54)	HG (n = 18)
**Age** (years)	**41.0** ± 1.8	**43.3** ± 2.7	**60.3** ± 1.8	**63.9** ± 1.6	**59.7** ± 1.7	**61.6** ± 1.4
**Gender Male** (N, %)	**4** (15%)	**1** (33%)	**16** (59%)	**6** (75%)	**35** (65%)	**12** (67%)
**BMI** (kg/m^2^)	**25.2** ± 1.0	**27.5** ± 3.7	**28.9** ± 0.9	**31.6** ± 2.7	**29.2** ± 0.9	**31.0** ± 1.2
**Obese** (BMI>30 kg/m^2^) (N, %)	**3** (11%)	**1** (33%)	**10** (37%)	**5** (63%)	**23** (43%)	**11** (61%)
**Glucose** (mmol/L)	**5.2** ± 0.1	**7.2** ± 0.3 *	**5.4** ± 0.1	**9.2** ± 0.7 ***	**5.4** ± 0.1	**9.9** ± 0.8 ***
**Hypertension** (N, %)	**0** (0%)	**0** (0%)	**23** (85%)	**6** (75%)	**35** (65%)	**9** (50%)
**Statin therapy** (N, %)	**0** (0%)	**0** (0%)	**22** (82%)	**6** (75%)	**40** (74%)	**11** (61%)
**Aspirin** (N, %)	**0** (0%)	**0** (0%)	**20** (74%)	**4** (50%)	**38** (70%)	**10** (56%) *
**Anti-platelets therapy** (N, %)	**0** (0%)	**0** (0%)	**12** (44%)	**3** (38%)	**34** (63%)	**11** (61%)
**ACE-Inhibitor** (N, %)	**0** (0%)	**0** (0%)	**20** (74%)	**5** (63%)	**35** (65%)	**11** (61%)

SA = patients with stable angina, ACS = patients with acute coronary syndrome, NG = normoglycemic patients, HG = hyperglycemic patients. Data are expressed as means ± standard error of the mean (SEM) and analyzed with Oneway ANOVA test with LSD Posthoc analysis. Chi-squared (χ^2^) analysis was performed to evaluate the differences between logistic data (gender, age distribution, obesity, hypertension, medication).

* p<0.05

*** p<0.001 HG vs. NG.

**Table 2:** Lipids, apolipoproteins and PON1 activity in sera of normoglycemic and hyperglycemic CAD patients and control subjects.

Parameter	Control (n = 30)		SA (n = 35)		ACS (n = 72)	
	NG (n = 27)	HG (n = 3)	NG (n = 27)	HG (n = 8)	NG (n = 54)	HG (n = 18)
**TC** (mmol/L)	**5.12** ± 0.17	**4.62** ± 0.36	**4.05** ± 0.17	**3.86** ± 0.43	**4.58** ± 0.19	**4.43** ± 0.33
**LDL-C** (mmol/L)	**3.29** ± 0.14	**2.61** ± 0.32	**2.17** ± 0.15	**1.96** ± 0.20	**2.86** ± 0.20	**2.30** ± 0.26
**HDL-C** (mmol/L)	**1.40** ± 0.06	**1.40** ± 0.12	**1.21** ± 0.07	**0.98** ± 0.06 *	**1.10** ± 0.05	**1.00** ± 0.10
**TG** (mmol/L)	**0.94** ± 0.08	**1.32** ± 0.17	**1.46** ± 0.14	**2.00** ± 0.49	**1.61** ± 0.12	**2.52** ± 0.34 **
**NEFA** (mmol/L)	**0.36** ± 0.03	**0.30** ± 0.10	**0.28** ± 0.02	**0.36** ± 0.10	**0.29** ± 0.02	**0.48** ± 0.08
**ApoA-I** (g/L)	**1.07** ± 0.06	**1.12** ± 0.15	**1.11** ± 0.06	**1.06** ± 0.08	**1.01** ± 0.05	**1.12** ± 0.06
**ApoB-100** (g/L)	**0.90** ± 0.05	**0.78** ± 0.00	**1.03** ± 0.07	**0.96** ± 0.11	**1.11** ± 0.05	**0.92** ± 0.10
**ApoE** (g/L)	**0.016** ± 0.001	**0.014** ± 0.002	**0.025** ± 0.002	**0.025** ± 0.008	**0.028** ± 0.003	**0.026** ± 0.003
**PON1 activity** (U/L)	**616.5** ± 75.9	**636.3** ± 484.4	**646.4** ± 77.0	**283.3** ± 54.3 ***	**479.5** ± 37.2	**381.3** ± 64.4

SA = patients with stable angina, ACS = patients with acute coronary syndrome, NG = normoglycemic patients, HG = hyperglycemic patients, TC = total cholesterol, TG = triglycerides, NEFA = non-esterified fatty acids. Data are expressed as means ± standard error of the mean (SEM) and analyzed with Oneway ANOVA test with LSD Posthoc analysis.

* p<0.05

** p<0.01

*** p<0.001 HG vs. NG.

**Fig 1:**
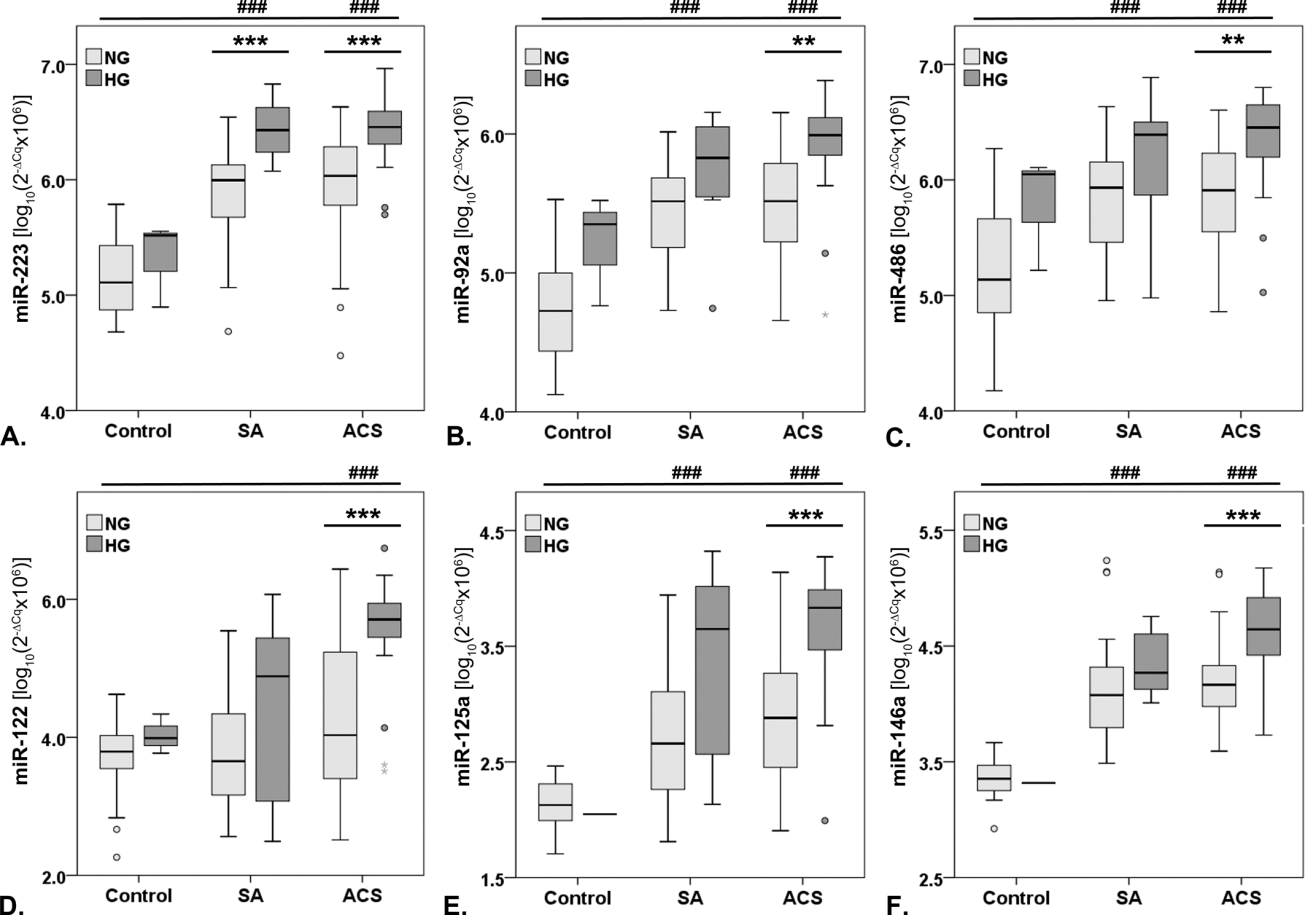
Boxplot distribution of the investigated miRNAs in sera. Levels of miR-223 (**A**), miR-92a (**B**), miR-486 (**C**), miR-122 (**D**), miR-125a (**E**), miR-146a (**F**) in sera from **Control** subjects and coronary artery disease (CAD) patients with stable angina (**SA**) or acute coronary syndrome (**ACS**), with/without hyperglycemia. Data are expressed as log-transformed 2^-ΔCq^ values, multiplied by 10^6^ ± standard error of the mean (** p<0.01, *** p<0.001 hyperglycemic (HG) vs. normoglycemic (NG), ^###^ p<0.001 SA or ACS vs. Control, Student T-test).

We have previously used the Pathway-focused Human CVD miScript miRNA PCR array [22], which includes validated CVD-related miRNAs to screen for those miRNAs modulated by CAD in sera. According to this initial screening, we selected the highest modulated miRNAs in ACS patients’ sera compared to control subjects’ sera, which were miR-223, miR-92a, miR-486 and miR-122. We then added miR-125a and miR-146a, based on preliminary data about hyperglycemia-related effects [25]. The analyzed miRNAs levels were significantly higher in all CAD patients’ sera compared to the controls’ sera (p<0.001, Fig 1A–1F), except for miR-122 in SA patients’ sera. The miRNAs mean levels showed no statistically significant differences between SA and ACS sera (Fig 1). Hyperglycemic ACS sera contained significantly higher levels of miR-223 (p<0.001), miR-92a (p = 0.001), miR-486 (p = 0.002), miR-122 (p<0.001), miR-125a (p<0.001) and miR-146a (p<0.001) compared to NG, ACS sera (Fig 1A–1F). In HG, SA sera compared to NG ones, only miR-223 levels were significantly increased (p<0.001, Fig 1A).

The bivariate parametric correlation analysis between log-transformed miRNAs levels and the anthropological and serum biochemical parameters of the subjects, expressed by the Pearson’s correlation coefficients and their corresponding p-values are presented in Table A in S1 File. The levels of all analyzed circulating miRNAs had a significant positive correlation with TG and glucose levels. The serum levels of miR-223, miR-92a, miR-486 and miR-146a were correlated positively with the age and BMI of the patients and control subjects and negatively with LDL-C levels. MiR-223 levels were positively correlated with apoE levels and negatively with HDL-C. All miRNAs levels, except for miR-223, were negatively correlated with PON1 activity. The levels of miR-92a and miR-486 correlated negatively with LDL-C/apoB-100 and PON1 activity/apoA-I. The levels of miR-122 were correlated positively with TG and NEFA levels and negatively with apoE levels.

**Fig 2:**
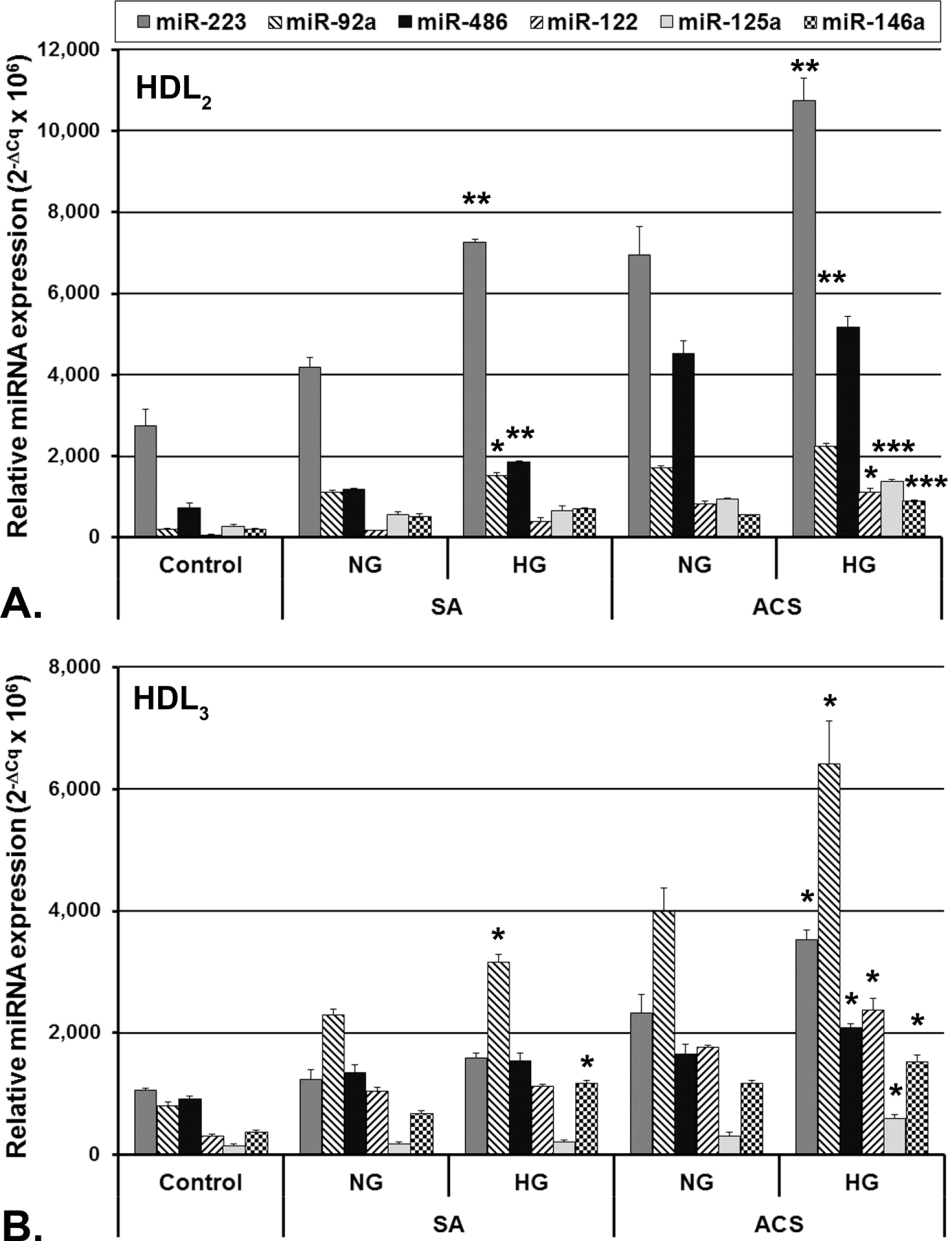
**Distribution of analyzed miRNAs in HDL subfractions: HDL**_**2**_ (**A**) and **HDL**_**3**_ (**B**) isolated by density gradient ultracentrifugation from pooled sera obtained from coronary artery disease (CAD) patients with stable angina (**SA**) and acute coronary syndrome (**ACS**), with/without hyperglycemia; the procedure was performed on 3 pools of sera from each group. Data are expressed as mean 2^-ΔCq^ values multiplied by 10^6^ ± standard error of the mean (* p<0.05, ** p<0.01, *** p<0.001 hyperglycemic (HG) vs. normoglycemic (NG), Student T-test).

HDL_2_ and HDL_3_ obtained after isopycnic density gradient ultracentrifugation of group-pooled sera were analyzed to determine the miRNAs content. The distribution of the selected miRNAs differed between HDL_2_ and HDL_3_ subfractions (Fig 2A and 2B). In HDL from all CAD patients, miR-223, miR-92a, miR-486 and miR-122 had the highest levels, miR-223 and miR-486 being the most abundant in HDL_2_ and miR-92a in HDL_3_ (Fig 2). Overall, all analyzed miRNAs had higher levels in HDL subfractions from patients’ sera compared to the ones from control subjects, except for miR-125a, whose levels in SA HDL_3_ did not differ significantly from control HDL_3_ (Fig 2). In HDL_2_, miR-223 and miR-486 showed the highest increase compared to control (Fig 2A), while in HDL_3_, miR-92a and miR-223 had the highest increase compared to control (Fig 2B).

HDL fractions from HG patients’ sera had higher levels of the selected miRNAs than NG ones, but only a few presented statistically significant differences (Fig 2, Table B in S1 File). In HDL_2_ from both groups of patients (Fig 2A), significantly higher levels of miR-223 and miR-92a were measured in HG compared to NG sera (Table B in S1 File). When comparing the values in HDL_2_ from the same group, miR-486 had significantly higher levels only in HG, SA versus NG, SA, while miR-122, miR-125a and miR-146a had significantly higher levels in HDL_2_ from HG, ACS compared to NG, ACS sera (Table B in S1 File).

In HDL_3_, significantly higher levels of miR-92a and miR-146a were measured in HG compared to NG sera (Fig 2B, Table B in S1 File). In HDL_3_ from HG, ACS sera, miR-223, miR-486, miR-122 and miR-125a had significantly higher levels compared to HDL_3_ from NG, ACS sera (Table B in S1 File).

### The expression of the processing machinery proteins and the production of miRNAs in human macrophages are increased by ACS sera

**Fig 3:**
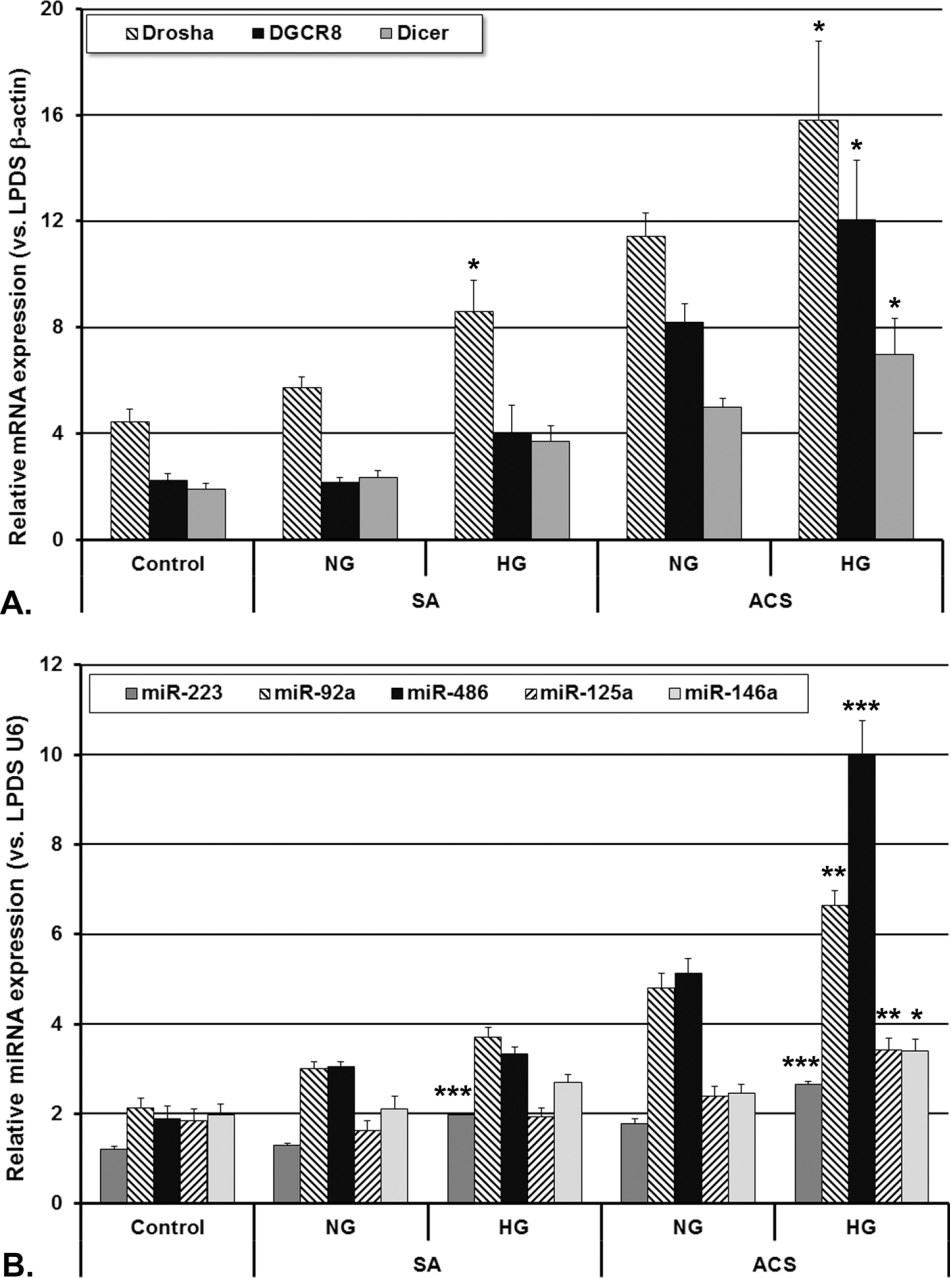
**Expression of Drosha, DGCR8 and Dicer mRNA (A) and of selected miRNAs (B) in human macrophages incubated with sera from CAD patients with stable angina (SA) or acute coronary syndrome (ACS), with/without hyperglycemia**. The expression level of each gene of interest was determined relative to β-actin, while the expression level of each miRNA was determined relative to snRNU6. Data are expressed as mean 2^-ΔΔCq^ values ± standard error of the mean. LPDS = human lipoprotein-deficient serum (* p<0.05, ** p<0.01, *** p<0.001 hyperglycemic (HG) vs. normoglycemic (NG), Student T-test).

To evaluate the effect of CAD patients’ sera on miRNAs production in human macrophages, we incubated the cells with sera from SA and ACS patients compared to control subjects. The gene expression of the processing machinery proteins (Drosha, DGCR8 and Dicer) and the selected miRNAs (miR-223, miR-92a, miR-486, miR-125a and miR-146a) levels are presented in Fig 3.

Overall, the gene expression of Drosha, DGCR8 and Dicer was increased in macrophages incubated with all CAD patients’ sera compared to control sera, having the highest values for ACS sera (3-fold for Drosha, 4.5-fold for DGCR8 and 3-fold for Dicer) (Fig 3A). The miR-223, miR-92a, miR-486, miR-125a and miR-146a levels measured after incubation of macrophages with SA and ACS sera were higher than those from control sera (Fig 3B). MiR-486 and miR-92a levels expressed the highest increase in macrophages incubated with ACS sera, compared to control and SA sera. In our experiments, miR-122 was undetectable in macrophages incubated with either serum.

The gene expression of Drosha, DGCR8 and Dicer was higher in cells incubated with HG, ACS sera compared to NG, ACS sera (p<0.05, Fig 3A). The HG, ACS sera induced an increase of all analyzed miRNAs levels (p<0.05) compared to NG, ACS sera-incubated macrophages, while in cells incubated with SA sera, only miR-223 levels were higher in HG compared to NG sera (p<0.001, Fig 3B), in good correlation with the increased gene expression of Drosha, DGCR8 and Dicer.

### High glucose increases miRNAs production in human macrophages exposed to CAD patients’ sera

To determine if the increase of glucose concentration itself may stimulate the effect of CAD sera on the production of miRNAs in human macrophages, we incubated the cells with sera from NG, SA and NG, ACS patients and with the respective sera with added glucose. To this purpose, we added 5.5 mM (100 mg/dl) glucose (+Gluc) to the NG patients’ sera before incubation with the cells to obtain a mean glucose concentration comparable with the one measured in the HG patients’ sera.

**Fig 4:**
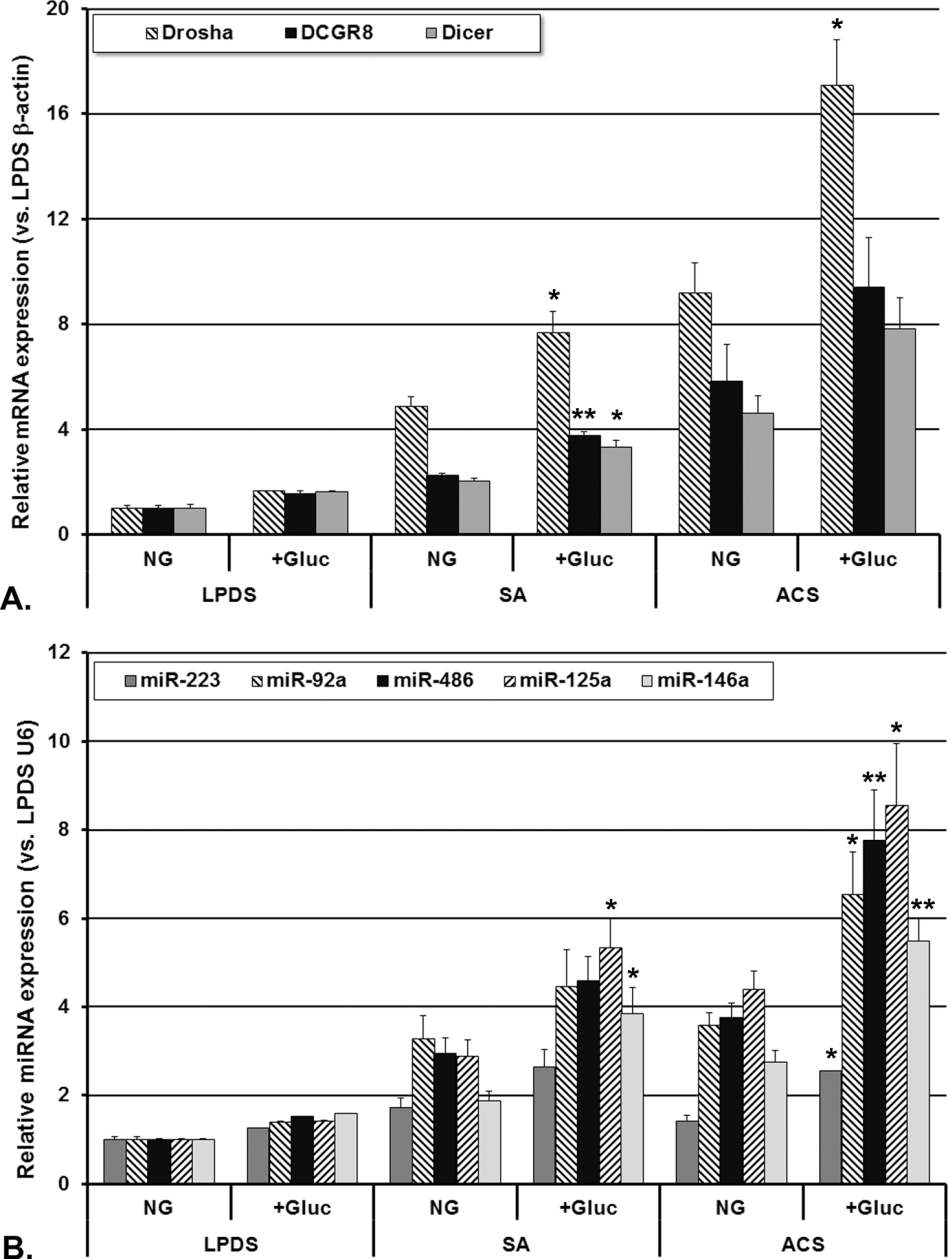
**Expression of Drosha, DGCR8 and Dicer mRNA (A) and of selected miRNAs (B) in human macrophages incubated with normoglycemic CAD patients’ sera with stable angina (SA) or acute coronary syndrome (ACS), with/without added glucose.** The expression level of each gene of interest was determined relative to β-actin, while the expression level of each miRNA was determined relative to snRNU6. Data are expressed as mean 2^-ΔΔCq^ values ± standard error of the mean. +Gluc = 5.5 mM (100 mg/dl) added glucose; LPDS = human lipoprotein-deficient serum (*p<0.05, **p<0.01 +Gluc vs. normoglycemic (NG), Student T-test).

The gene expression of the processing machinery components (Drosha, DGCR8 and Dicer) and the production of the selected miRNAs (miR-223, miR-92a, miR-486, miR-125a and miR-146a) are presented in Fig 4. The gene expression of Drosha, DGCR8 and Dicer was upregulated in cells incubated with either sera with added glucose compared to the NG sera (an average of 2 fold, p<0.05, Fig 4A).

The sera with the added glucose (+Gluc) determined an increase of all the studied miRNAs production in macrophages (Fig 4B), the most significant increase being measured in cells incubated with ACS+Gluc sera (average 2 fold, p<0.05). We also observed that miR-125a expressed the highest increase in macrophages incubated with ACS+Gluc sera compared to NG, ACS sera (2 fold, p<0.05). Moreover, only miR-125a and miR-146a levels expressed a significant increase in human macrophages exposed to SA+Gluc sera compared to NG, SA sera, in good correlation with the increase of the gene expression of Drosha, DGCR8 and Dicer.

## Discussion

The new data about the effect of hyperglycemia on miRNAs obtained in the present study are: (i) in hyperglycemic ACS sera, miR-223, miR-92a, miR-486, miR-122, miR-125a and miR-146a levels are increased compared to the normoglycemic ACS sera; (ii) in HDL from hyperglycemic ACS compared to normoglycemic sera, miR-223, miR-92a, miR-486 are increased and statistically different between ACS and SA patients; (iii) ACS sera induce an increase of Drosha, DGCR8 and Dicer expression and of miR-223, miR-92a, miR-486, miR-125a and miR-146a production in human macrophages; (iv) the hyperglycemic sera augments the effects observed in human macrophages exposed to normoglycemic sera, mainly in the case of ACS sera.

In sera, the selected miRNAs levels were higher in CAD patients compared to control subjects, but showed no significant differences between SA and ACS patients. However, in HG, ACS sera the levels of miR-223, miR-92a, miR-486, miR-122, miR-125a and miR-146a were higher compared to the ones in NG, ACS sera. In HG, SA compared to NG, SA sera, only miR-223 presented higher levels. To date, there are only few studies to investigate the circulating miRNAs in hyperglycemic CAD patients’ sera. It was reported that circulating miR-223 levels may predict cardiovascular death over a 4 years period in symptomatic CAD patients [26]. Circulating miR-92a levels were reported to be increased in the sera of diabetic SA patients compared to non-diabetic ones [27, 28], in good agreement with our results. In contrast, Liu et al. reported that the levels of circulating miR-92a are lower in CAD patients than in control subjects and positively correlated with HDL and apoA-I levels [29]. Circulating levels of miR-146a were reported to be markedly elevated in ACS patients [30], in good agreement with our data.

In our study, the parametric correlations show that miR-92a and miR-486 correlate negatively with two risk factors for CAD: small dense LDL (expressed as LDL-C/apoB-100) and dysfunctional HDL (expressed as PON1 activity/apoA-I). This suggests that when small dense LDL and dysfunctional HDL coexist, miR-92a and miR-486 levels increase, confirming our previous results that show a preferential association of miR-92a and miR-486 in vulnerable CAD patients [22].

In addition to our previous observations [22] and those of Vickers et al. [8] and Wagner et al. [11], we report here that HDL subfractions from ACS patients’ sera have higher amounts of miR-223 and miR-486 in HDL_2_, and miR-92a in HDL_3_, compared to the respective HDL subfractions in Control and SA patients’ sera, their levels being further augmented in hyperglycemic ACS sera.

To our knowledge, this is the first study to investigate the effect of CAD patients’ sera on the miRNAs expression and processing machinery in human macrophages. We report that the exposure of human macrophages to ACS sera induces the increase of the intracellular miR-223, miR-92a, miR-486, miR-125a and miR-146a levels in human macrophages, the highest levels being observed for miR-486 and miR-92a. This increase could be attributed to several factors: an upregulation of the processing machinery proteins (Drosha, DGCR8 and Dicer) induced by factors from the patients’ sera, the transfer of circulating miRNAs from serum HDL or other extracellular vesicles to the cells, or the upregulation of the selected miRNAs expression by other serum factors. We report for the first time that miR-122 is undetectable in human THP-1 macrophages, in all analyzed culture conditions.

To date, there are few reports regarding the CAD-induced modulation of miRNAs production in human macrophages. Lai et al. showed that miR-92a induces the reduction of the production of inflammatory cytokines in macrophages by a Toll-like receptor (TLR)-mediated mechanism [31]. In turn, miR-223 upregulation reduces TLR4 signaling [32] and the activation of the signal transducer and activator of transcription 3 (STAT3) [33], thus suppressing the production of pro-inflammatory cytokines in macrophages [34]. It is known that the expression of miR-146a/b is increased in human atherosclerotic plaques [35], probably as a protective feedback mechanism, being known that miR-146 overexpression downregulates NF-κB activation that in turn reduces atherosclerosis [36]. Pro-inflammatory M1 macrophages exhibit an increased expression of miR-125a [37], which could also be a protective feedback mechanism, because the inhibition of the endogenous miR-125a levels in THP-1 macrophages increases the secretion of inflammatory cytokines and the expression of macrophage scavenger receptors resulting in increased lipid uptake [38].

The addition of glucose to the NG sera upregulated Drosha, DGCR8 and Dicer expression and further increased the miRNAs production in macrophages exposed to patients’ sera. To our knowledge, these are the first data reporting the effect of increased glucose on the miRNAs production in human macrophages. We aimed to correlate the data obtained *in vitro*, by exposing human macrophages to CAD patients’ sera with the measured levels of the selected miRNAs in the patients’ sera. The observed increase of miRNAs production in macrophages is in accordance with the increased levels of specific miRNAs in sera, suggesting either an uptake of miRNAs from sera, or an upregulation of the cellular miRNAs production. The addition of glucose to normoglycemic sera (to mimic the existing concentration in hyperglycemic ones) induces both an increase of the miRNAs (miR-125a, miR-486, miR-92a, miR-146a, and miR-223) levels and of their processing machinery proteins (Drosha, DGCR8 and Dicer).

In conclusion, the hyperglycemia in serum is correlated with the increased levels of miR-223, miR-92a, miR-486 in sera and HDL, these miRNAs associated with HDL being able to discriminate between ACS and SA patients. Exposure of human macrophages to ACS sera compared to SA sera determines an increase of the production of miR-223, miR-92a, miR-486, miR-125a and miR-146a by the upregulation of Drosha, DGCR8 and Dicer expression, this effect being augmented by the increase of sera’s glucose concentration.

## Supporting Information

S1 File Supporting information file for the manuscript. This file contains Table A and Table B.
(DOCX)

S2 File Supporting data for the manuscript. This file contains individual data points behind the means, medians and variance measures presented in the results, tables and figures.
(XLS)
