# Supplementary material for: Hyperglycemia Determines Increased Specific MicroRNAs Levels in Sera and HDL of Acute Coronary Syndrome Patients and Stimulates MicroRNAs Production in Human Macrophages
Source: PLoS One. 2016 Aug 12;11(8):e0161201. doi: 10.1371/journal.pone.0161201 (PMC4982674; doi:10.1371/journal.pone.0161201)
Supplement: S1 File — This file contains Table A and Table B. (DOCX) [file pone.0161201.s001.docx]

**ELECTRONIC SUPPLEMENTARY MATERIAL**

***PLOS One***

**Hyperglycemia determines increased specific microRNAs levels in sera and HDL of acute coronary syndrome patients and stimulates microRNAs production in human macrophages**

Natalia Simionescu ^1,2 ¶^, Loredan S. Niculescu ^1 ¶^, Mihaela G. Carnuta ^1^, Gabriela M. Sanda ^1^, Camelia S. Stancu ^1^, Andreea C. Popescu ^3^, Mihaela R. Popescu ^3^, Adelina Vlad ^3,4^, Doina R. Dimulescu ^3^, Maya Simionescu ^1^, Anca V. Sima ^1 *^

^1^ Lipidomics Department, Institute of Cellular Biology and Pathology “Nicolae Simionescu” of the Romanian Academy, 8 B.P. Hasdeu Street, Bucharest 050568, Romania

^2^ Centre of Advanced Research in Bionanoconjugates and Biopolymers, “Petru Poni” Institute of Macromolecular Chemistry, 41A Grigore Ghica Voda Alley, Iasi 700487, Romania

^3^ Cardiology Clinic, Elias University Emergency Hospital, 17 Marasti Blvd, Bucharest 011461, Romania

^4^ Physiology Department, “Carol Davila” University of Medicine and Pharmacy, 37 Dionisie Lupu Street, Bucharest 020021, Romania

^¶^ *These authors contributed equally to this work.*

^*^ Corresponding author

Anca V. Sima, Ph.D., Member of the Romanian Academy

Head, Lipidomics Department

Institute of Cellular Biology and Pathology “N. Simionescu” of the Romanian Academy

8, B.P. Hasdeu Street, Bucharest 050568, Romania

E-mail: anca.sima@icbp.ro; Phone: +40.21.319.4518; Fax: +40.21.319.4519

**SUPPLEMENTARY TABLES**

**Table A. Pearson’s parametric correlation coefficients of serum miRNAs levels with some parameters of the studied subjects**

|  | **miR-223** | **miR-92a** | **miR-486** | **miR-125a** | **miR-146a** | **miR-122** |
| --- | --- | --- | --- | --- | --- | --- |
| **Age** | 0.372  *** | 0.291  ** | 0.230  ** | 0.099 | 0.323  *** | -0.002 |
| **BMI** | 0.314  ** | 0.282  ** | 0.227  * | 0.200  * | 0.357  *** | 0.113 |
| **Glucose** | 0.415  *** | 0.386  *** | 0.349  *** | 0.435  *** | 0.394  *** | 0.357  *** |
| **TG** | 0.357  *** | 0.328  *** | 0.229  ** | 0.271  ** | 0.223  * | 0.194  * |
| **NEFA** | -0.027 | 0.140 | 0.170  * | 0.317  ** | 0.162 | 0.308  *** |
| **TC** | -0.171 | -0.164 | -0.162 | -0.045 | -0.262  ** | 0.018 |
| **LDL-C** | -0.262  ** | -0.252  ** | -0.252  ** | -0.105 | -0.374  *** | 0.004 |
| **HDL-C** | -0.388  *** | -0.204  * | -0.085 | -0.181 | -0.190 | -0.083 |
| **ApoA-I** | -0.157 | -0.089 | -0.067 | -0.109 | -0.235  * | -0.040 |
| **ApoB-100** | 0.121 | -0.078 | -0.077 | -0.216  * | -0.204 | -0.281  ** |
| **ApoE** | 0.255  ** | 0.099 | 0.039 | -0.058 | 0.086 | -0.186  * |
| **PON1 activity** | -0.148 | -0.343  *** | -0.365  *** | -0.345  *** | -0.226  * | -0.390  *** |
| **LDL-C/ApoB-100** | -0.282  * | -0.319  ** | -0.347  ** | -0.086 | -0.363  ** | 0.083 |
| **HDL-C/ApoA-I** | -0.293  ** | -0.160 | -0.076 | -0.183 | 0.034 | -0.192  * |
| **PON1/ApoA-I** | -0.038 | -0.243  ** | -0.274  ** | -0.306  ** | -0.063 | -0.366  *** |

Data represents the Pearson’s correlation coefficients and their associated statistical significance (p-values);

* p<0.05; ** p<0.01; *** p<0.001.

**Table B. Fold change values of mean miRNAs levels in HDL subfractions from normoglycemic versus hyperglycemic CAD patients’ sera**

| **HDL subfraction** | **MicroRNA** | **SA** | **ACS** |
| --- | --- | --- | --- |
| **HDL_2_** | **miR-223** | **1.7** ** | **1.5** ** |
|  | **miR-92a** | **1.4** * | **1.3** ** |
|  | **miR-486** | **1.6** ** | **1.1** |
|  | **miR-122** | **2.3** | **1.3 *** |
|  | **miR-125a** | **1.2** | **1.5** *** |
|  | **miR-146a** | **1.4** | **1.6** *** |
| **HDL_3_** | **miR-223** | **1.3** | **1.5** * |
|  | **miR-92a** | **1.4 *** | **1.6** * |
|  | **miR-486** | **1.2** | **1.3** * |
|  | **miR-122** | **1.1** | **1.4** * |
|  | **miR-125a** | **1.2** | **1.9** * |
|  | **miR-146a** | **1.7** * | **1.3** * |

SA=patients with stable angina, ACS=patients with acute coronary syndrome. Data are expressed as fold change values of mean miRNAs levels in HDL subfractions from normoglycemic (NG) versus hyperglycemic (HG) patients’ sera and their statistical significance (p-values, Student T-test). * p<0.05; ** p<0.01; *** p<0.001 HG vs. NG.
